# Case report: Non-linear evolution of oxytocin informs YBOCS changes post-DBS of the bed nucleus of the stria terminalis for treatment resistant OCD

**DOI:** 10.3389/fpsyt.2024.1473797

**Published:** 2025-01-27

**Authors:** Jean-Baptiste Belge, Vincent Geenen, Anne L. Salado, Bruno Kaschten, Didier Martin, Gabrielle Scantamburlo

**Affiliations:** ^1^ Department of Psychiatry, Liège University Hospital, University of Liège, Liège, Belgium; ^2^ Psycho-Neuro-Endocrinology Unit, University of Liège, Liège, Belgium; ^3^ GIGA Research Institute, GIGA-Immunity, Inflammation and Infection (GIGA-I3), University of Liège, Liège, Belgium; ^4^ Department of Neurosurgery, Liège University Hospital, University of Liège, Liège, Belgium

**Keywords:** DBS (deep brain stimulation), OCD (obsessive-compulsive disorder), oxytocin, non-linear, neuromodulation

## Abstract

**Introduction:**

Obsessive-compulsive disorder (OCD) is a challenging neuropsychiatric condition with a subset of patients remaining refractory to conventional treatments. Deep brain stimulation (DBS) of the bed nucleus of the stria terminalis (BNST) has shown promise for severe, treatment-resistant OCD. This case report examines the relationship between plasma oxytocin levels and OCD symptom severity following BNST-DBS.

**Methods:**

A 36-year-old patient with long-standing, treatment-resistant OCD underwent stereotactic implantation of DBS electrodes at the BNST. Postoperative assessments included OCD symptom severity using the Yale-Brown Obsessive Compulsive Scale (YBOCS) and plasma oxytocin levels, measured at 12 time points over three years. Longitudinal and correlational analyses were performed using linear and polynomial regression models.

**Results:**

Non-linear trends in oxytocin levels were identified, with polynomial regression revealing a significant quadratic term, suggesting a parabolic trend. Strong positive correlations were found between changes in oxytocin levels and YBOCS total, obsession, and compulsion scores.

**Conclusion:**

The findings suggest a significant non-linear evolution of oxytocin levels and a positive correlation with OCD symptom changes following BNST-DBS. Oxytocin levels could serve as a biomarker for DBS efficacy if this finding is replicated in larger studies.

## 1 Introduction

Obsessive-compulsive disorder (OCD) poses a significant challenge in neuropsychiatric care. While conventional treatments are effective, a subset of patients remains refractory to all interventions (1, 2). Deep brain stimulation (DBS) has emerged as a promising avenue for treating severe, treatment-resistant OCD (3). Early applications of DBS in OCD treatment focused on regions such as the anterior limb of the internal capsule (ALIC) (4, 5). Subsequent research has explored alternative targets within and beyond the cortico-striato-thalamo-cortical circuitry, including the bed nucleus of the stria terminalis (BNST). A vast body of research, has demonstrated the efficacy of DBS of the BNST (6–9). Moreover, in a long-term follow-up study the BNST appeared to be a more effective stimulation target compared to ALIC for alleviating OCD symptoms (10). The neurobiological mechanisms underlying the positive effects of BNST stimulation remain uncertain. From a neurochemical perspective, the BNST is rich in oxytocin (OT) receptors (11). OT, a neuropeptide implicated in OCD (12), plays a crucial role in modulating inhibitory brain circuits (13). This is particularly relevant as OCD is characterized by an inhibitory deficit in various brain regions (14, 15). However, the complex relationship between BNST-DBS, OT and OCD symptomatology has yet to be explored. This case report aimed to investigate for the first time the longitudinal relationship between changes in plasma OT levels and OCD symptom severity as measured with the YBOCS (Yale-Brown Obsessive Compulsive Scale), following BNST-DBS. By elucidating the potential role of oxytocin in mediating DBS-induced symptom improvement, this report seeks to contribute to a deeper understanding of the neurobiological mechanisms underlying BNST-DBS efficacy in OCD treatment.

## 2 Methods

### 2.1 Participant

The 36-year-old patient had OCD since adolescence, starting at 17, characterized by obsessions and compulsions related to symmetry accompanied by checking compulsions, collecting, ordering, arranging, and repeating rituals where doubt was omnipresent. The main treatment at the time of surgery was paroxetine (60 mg/day). Therapies combining antidepressants, antipsychotics, benzodiazepines, cognitive behavioral therapy and electroconvulsive therapy had proven ineffective. A neurosurgical intervention was performed in two stages on the same day, involving stereotactic bilateral implantation of two electrodes (Activa RC) at the level of the BNSTand subsequent tunneling of extensions to the neurostimulator implanted subcutaneously in the right hypochondrium. The procedure was without complications, and postoperative brain scans confirmed the correct electrode positioning.

### 2.2 Psychometric and biological measurements

The psychometric and biological data were collected by a psychiatrist specialized in OCD and DBS, with time points aligned to clinical evaluations of DBS efficacy. The data collection took place at the interventional Psychiatry Unit of the University Hospital of Liège.

Blood samples were drawn at 08:00 h in the morning and centrifuged within 2 hours, with the serum immediately frozen and stored at -181°C until analysis. OT levels were measured using a double antibody radioimmunoassay, with intra-assay variability of 3.41% and interassay variability of 2.84%, and a sensitivity of 1 pmol/l.

The patient was first stimulated 11 days after surgery, and data were collected at 12 separate time points: T-1: (Just before surgery), T0 (day of first stimulation), T1 (T0 + 6days), T2 (T0 + 19days), T3 (T0 + 29 days), T4 (T0 + 63 days), T5 (T0 + 3 months), T6 (T0 + 9 months), T7 (T0 + 1year), T8 (T0 + 603 days), T9 (T0 + 616 days), T10 (T0 + 693 days), T11 (T0 + 2 years), T12 (T0 + 3years). Stimulator variables (amplitude left, amplitude right, frequency left, frequency right, pulse left, pulse right) were measured on all time points. YBOCS (Yale-Brown Obsessive Compulsive Scale) scores were measured on T-1, T1, T3, T4, T5,T6,T7,T8,T9,T10, T11, T12, Plasma Oxytocin levels were measured on T0,T3,T4,T8, T9,T10.

For the longitudinal analysis of change over time, all available data points were used for each variable. For the correlational analysis between longitudinal changes only complete data sets were considered (T3,T4,T8, T9,T10). As during the treatment only adaptations of the DBS amplitude were done, only these were taken into account for longitudinal and correlational analysis. All statistical analysis were carried out in Matlab R2023a.

## 3 Results

The statistical analysis was designed to explore longitudinal trends and relationships between key variables over time, focusing on potential linear and non-linear patterns. This is in important as in biology, feedback mechanisms and biological variation often give way to non-linear relationships (16, 17).

Non-linear trends were visually assessed by plotting all variables over time. Visual inspection identified potential non-linearity only in plasma oxytocin.

To analyze the longitudinal changes of the YBOCS scores and the bilateral amplitudes over time, we generated a corresponding time vector for each observation and fitted a linear regression model employing the film function in Matlab. The intercept of 19.47 (p = 0.052) and slope of -1.03 (p = 0.476) suggest a slight but non-significant decrease in YBOCS-t scores over time. This was also the case for the YBOCS-o (obsession subscale) scores, with an intercept of 9.20 (p = 0.028) and a slope of -0.44 (p = 0.385), and the YBOCS-c (compulsion subscale) scores, with an intercept of 9.94 (p = 0.023) and a slope of -0.54 (p = 0.306). There was a significant increase over time in the bilateral amplitude: left electrode, intercept of 3.45 (p < 0.001) and slope of 0.056 (p = 0.002), and right electrode, intercept of 3.45 (p < 0.001) and slope of 0.056 (p = 0.002).

**Figure 1:**
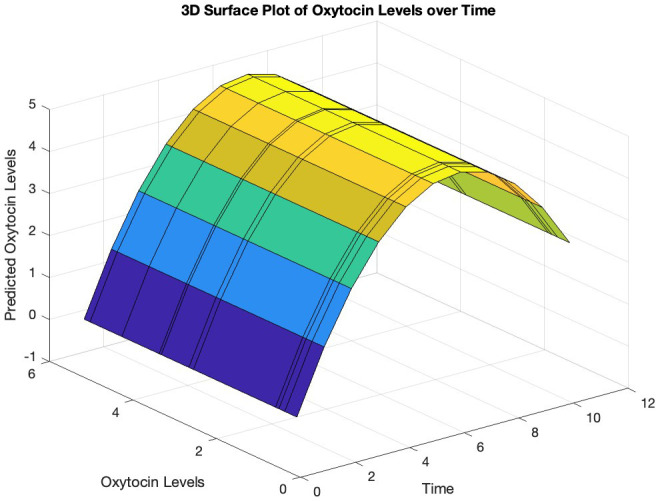
Predicted trajectory of oxytocin levels over time. The surface plot illustrates the relationship between the predicted oxytocin levels (y-axis) and time (x-axis), with the z-axis representing the measured oxytocin levels based on the polynomial regression fit. The curved surface suggests a non-linear relationship between oxytocin levels and time.

**Figure 2:**
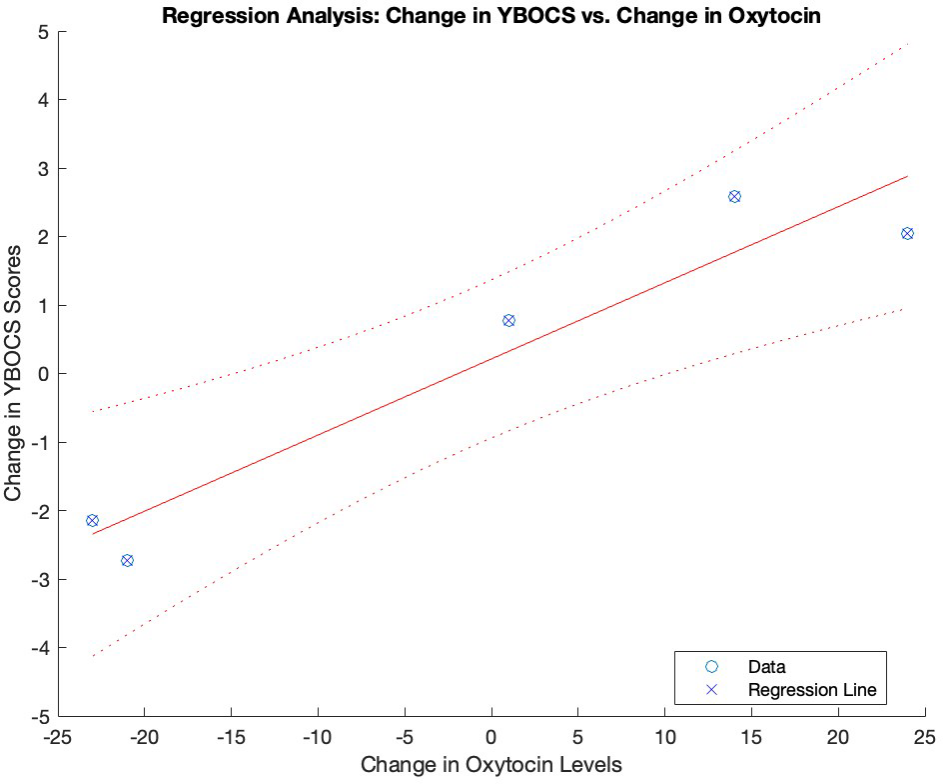
Association between changes in oxytocin levels and YBOCS scores over time. Graphical representation of the regression analysis between the changes in oxytocin levels and YBOCS scores over time. YBOCS, Yale-Brown Obsessive- Compulsive scale..

To investigate the non-linearity in oxytocin levels over time, we conducted the Ramsey RESET test. After fitting a linear regression model to the oxytocin data, we added squared and cubic terms. The analysis yielded a significant F-statistic of 9.95 (p = 0.009), indicating that the augmented model fit the data significantly better, suggesting a non-linear relationship between oxytocin levels and time. Subsequently, polynomial regression analysis was performed using the polyfit function, revealing a parabolic trend in oxytocin levels over time with the quadratic term coefficient = -0.139 (p = 0.013) (**Figures 1**, **2**). An analysis of variance was performed to test the significance of the polynomial model, revealing a significant overall model fit (F = 7.02, p = 0.017).

To investigate the correlations between changes over time, we computed the deltas of the variables over time. Matlab’s corr function was utilized to compute correlation coefficients and their corresponding p-values. The results revealed highly significant positive correlations between changes over time in oxytocin levels and YBOCS total scores (r = 0.957, p < 0.001), as well as YBOCS-o scores (r = 0.957, p < 0.001) and YBOCS-c scores (r = 0.956, p < 0.001). No significant correlation between changes over time in YBOCS-t scores and changes in amplitude-left (r = -0.079, p = 0.829) or amplitude-right (r = -0.079, p = 0.829) was found. Finally, there was no significant correlation between changes over time in amplitude-left and changes in oxytocin levels (r = -0.079, p = 0.829), or changes in amplitude-right and changes in oxytocin levels (r = -0.079, p = 0.829). There was no interaction between changes in pharmacological treatment dosage and oxytocin levels (r = 0.410, p = 0.239).

## 4 Discussion

After DBS of the BNST for OCD we observed a significant non-linear evolution of OT levels and a positive correlation between changes in the YBOCS scale and oxytocin levels over time. To our best knowledge this is the first time that such findings are being reported.

Several animal studies provide compelling evidence for the involvement of OT in OCD (12, 18). In humans elevated cerebrospinal fluid levels of oxytocin have been correlated with OCD severity (19). Similar results were found by 20 who reported a positive correlation between baseline plasma oxytocin and YBOCS scores in untreated OCD patients compared to healthy controls. Further, investigating oxytocin levels in the cerebrospinal fluid of children/adolescents with OCD before and after clomipramine treatment Altemus et al. (21) noted an overall increase in oxytocin levels but a parodoxical negative correlation with clinical response.

Oxytocin plays a crucial role in modulating fear and anxiety-related behaviors (22, 23). At the cellular level, oxytocin exerts a potent influence on various inhibitory neurocircuits especially by increasing GABAergic interneuron activity (13, 24–26). For instance, recent research demonstrated that optogenetic activation of hypothalamic OT neurons triggers the activation of a local GABAergic circuit (23). This holds particular significance for OCD, a disorder characterized by deficient inhibitory control and hyperactivity in key brain regions (14). Studies have shown hyperactivity in areas like the supplementary motor area (SMA) and anterior cingulate cortex (ACC) in OCD, potentially stemming from inadequate inhibitory control (15). Neurochemical investigations using 7-Tesla proton magnetic resonance spectroscopy revealed imbalances in excitatory and inhibitory neurotransmission, evidenced by altered glutamate and GABA levels in the ACC and SMA among OCD patients compared with healthy controls (27). Notably, this could also explain the efficacy of low-frequency, inhibitory repetitive transcranial magnetic stimulation targeting the SMA for pharmacoresistant OCD (28, 29).

Fascinatingly, the BNST exhibits one of the highest expression levels of oxytocin receptors (OTR) in the rodent brain, as evidenced by numerous studies (11, 30). BNST neurons are predominantly GABAergic and are intricately interconnected through an extensive intrinsic inhibitory network (31, 32). In the BNST oxytocin appears to enhance the intrinsic excitability and spontaneous firing frequency of regular spiking neurons, thereby augmenting inhibitory synaptic transmission (33). This finding holds particular intrigue, given that animal models of OCD suggest hyperactivity in the BNST (18). It’s thus tempting to speculate that in OCD, oxytocin’s inhibitory effects serve a compensatory role by attempting to attenuate the heightened activity in the BNST or in other brain regions. This hypothesis could explain the elevated oxytocin levels observed in OCD individuals (20) and the subsequent decline in oxytocin levels among those who respond favorably to therapy, where excessive oxytocin levels may no longer be necessary as inhibitory function gradually normalizes (21). Finally the fact that epigenetic studies have suggested a link between OTR hypermethylation and OCD symptom severity reinforce the idea that high OT levels may serve a compensatory mechanism with higher symptom severity when this mechanism is dysfunctional (34).

Finally, The mechanisms underlying DBS of the BNST in OCD remain elusive. Typically, high-frequency electrical stimulation applied to specific targets within subcortical structures inhibits local neuronal activity by activating GABAergic afferents in the stimulated nucleus (35–37). Given the abundance of GABAergic cells in the BNST, it is plausible that DBS of this region activates these inhibitory interneurons. As a matter of fact, high-frequency DBS of the BNST has been observed to reduce oscillatory theta band activity in both the BNST and the frontal cortex, suggesting an enhancement in inhibitory functions (38, 39).

We propose that the observed non-linear evolution of oxytocin levels and the positive association with changes in the YBOCS scale over time could be attributed to DBS reinforcing inhibitory neural networks. DBS, in this case targeting the BNST, stimulates inhibitory GABAergic interneurons, gradually strengthening intrinsic inhibitory networks. The fluctuations in oxytocin levels may serve as an indicator of the effectiveness of DBS therapy in restoring inhibitory neural network function. A decrease of oxytocin levels might signify the attainment of an inhibitory neural network self-sufficiency, no longer reliant on elevated oxytocin levels to maintain proper functioning. Initially, high levels of oxytocin may be necessary to compensate for deficient inhibitory circuitry. However, as DBS therapy effectively reinforces the inhibitory network, the need for high oxytocin levels diminishes. Consequently, oxytocin levels gradually decrease over time as the inhibitory circuitry becomes more efficient and the clinicial symptoms improve. The lack of a direct correlation between changes in DBS parameters and symptom improvement or OT levels could suggest that the association between changes in YBOCS scores and oxytocin levels is more indirect and relates more to the restauration of inhibitory network efficacy. Finally, the lack of a significant association between DBS parameters and YBOCS scores could be due to the limited power of this single case study.

Although this is only a single case, requiring caution in overinterpreting the data, when replicated in much larger sample sizes, the potential of oxytocin levels as a reliable biomarker for monitoring the efficacy of BNST-DBS in treating OCD could be significant. Larger studies would allow for more robust statistical analysis, helping to clarify whether changes in oxytocin levels correlate consistently with clinical improvements, and whether oxytocin could serve as an early indicator of treatment response. If confirmed, this could provide valuable insights into the neurobiological mechanisms of BNST-DBS and offer a non-invasive method for tracking treatment progress, ultimately improving personalized approaches to OCD management.

## Data Availability

The datasets presented in this article are not readily available because Ethical restrictions and refusal by the patient. Requests to access the datasets should be directed to jean-baptiste.belge@chuliege.be.
